# The functional and clinical significance of nucleoporin NUP153 across human cancers: a systematic study based on multi-omics analysis and bench work validation

**DOI:** 10.3389/fimmu.2025.1613688

**Published:** 2025-06-18

**Authors:** Youfu He, Qing Wang, Zhaorui Wang, Menghui Duan, Yu Zhou, Jing Huang, Qiang Wu, Feichang Wu, Chen Li

**Affiliations:** ^1^ Medical College, Guizhou University, Guiyang, Guizhou, China; ^2^ Department of Cardiology, Guizhou Provincial People’s Hospital, Guiyang, Guizhou, China; ^3^ Department of Colorectal Surgery, the First People’s Hospital of Zhengzhou, Zhengzhou, Henan, China; ^4^ Translational Medicine Research Center, The Fifth Clinical Medical College of Henan University of Chinese Medicine (Zhengzhou People’s Hospital), Zhengzhou, Henan, China; ^5^ Department of Critical Care Medicine, The First Hospital of China Medical University, Shenyang, Liaoning, China; ^6^ Department of Oncology, Gaozhou People’s Hospital, Maoming, Guangdong, China; ^7^ Department of Pharmacy, the First Affiliated Hospital of Guangxi Medical University, Nanning, Guangxi, China

**Keywords:** NUP153, gastric cancer, tumour immune microenvironment, chemotherapy sensitivity, prognostic biomarker

## Abstract

**Background:**

Cancer is a group of highly heterogeneous malignant diseases, and early diagnosis plays a crucial role in improving patient outcomes. Nucleoporins, including Nucleoporin (NUP)153, are involved in key cellular processes such as nucleocytoplasmic transport and cell cycle regulation. However, the role of NUP153 in cancer, especially its expression patterns, mutations, diagnostic value, and relationship with the tumour immune microenvironment, remains insufficiently explored.

**Methods:**

This study analysed NUP153 expression data from public databases such as TCGA and GTEx. Expression differences between tumour and normal tissues were assessed using Wilcoxon signed-rank tests. Gene set enrichment analysis (GSEA) was used to identify the biological functions and pathways related to NUP153. The relationship between NUP153 expression and immune cell infiltration was evaluated using the TIMER database, while drug sensitivity data were obtained from the GDSC and CTRP databases. Additionally, NUP153 expression in gastric cancer tissues was validated using immunohistochemistry and RT-qPCR.

**Results:**

NUP153 showed significant expression variation across cancers, with high levels in cholangiocarcinoma, colorectal cancer, and head and neck squamous cell carcinoma. In gastric cancer, NUP153 was markedly upregulated compared to adjacent non-cancerous tissues. High NUP153 expression was linked to tumour-associated macrophage infiltration and correlated with poor prognosis in some cancers like Kidney Renal Papillary Cell Carcinoma and Sarcoma. Drug genomics analysis revealed that NUP153 expression predicted chemotherapy resistance, with imatinib and 4.5-dianilinophthalimide showing potential for inhibition in multiple cancers. Single-cell analysis and spatial transcriptomics further revealed that NUP153 expression drives proliferative states in mucus-producing cells in gastric cancer, and its expression was associated with immune cell infiltration patterns, particularly neutrophil and macrophage distribution in the tumour microenvironment.

**Conclusion:**

Our findings indicate that NUP153 is a critical factor in multiple cancers, especially gastric cancer, where its elevated expression holds promise as a diagnostic and prognostic biomarker. The results indicate that NUP153 plays a key role in modulating the immune microenvironment and driving tumour progression, positioning it as a potential target for future therapeutic interventions. However, additional studies are required to elucidate the specific molecular mechanisms underlying NUP153’s function in cancer and to explore its clinical applicability.

## Introduction

Cancer is a group of highly heterogeneous malignant tumours. According to a 2019 report by the World Health Organization, it has become the leading or second leading cause of death in 112 out of 183 countries for individuals under 70 years old (1). In high-income countries, the most common cancers among adults include lung cancer, colorectal cancer, female breast cancer, melanoma, and prostate cancer (2). Globally, cancer mortality rates have been declining, primarily due to improved risk factor prevention, expanded early detection programmes, including screening, and advances in treatment interventions (3). Given sufficient research showing that early diagnosis can significantly reduce the mortality risk of breast cancer, cervical cancer, colorectal cancer, lung cancer, and prostate cancer (4, 5), optimizing early cancer diagnosis has become a key research focus.

The nuclear pore complex (NPC) is a large, highly organised structure embedded in the double membrane of the nuclear envelope. It is made up of three main parts: the cytoplasmic ring, which connects to filaments extending into the cytoplasm; the nuclear ring, which is linked to a basket-like structure on the nuclear side; and a central scaffold that spans the space in between. Together, these components enable the selective, two-way movement of molecules between the nucleus and the cytoplasm (6). It consists of approximately 30 proteins, known as nucleoporins, which together form the structural and functional framework of the NPC. Through this assembly, the complex acts as the sole gateway for the regulated, bidirectional transport of macromolecules between the nucleus and the cytoplasm (7), a process that plays a critical role in maintaining cellular homeostasis. Disruption of this transport mechanism has been increasingly linked to cancer progression, as the invasion and metastasis of tumours are closely associated with abnormal nucleocytoplasmic exchange. Several studies have revealed the relationship between NPCs and cancers, such as Nucleoporin(NUP)62 (8), NUP98 (9), NUP214 (10), among others. Beyond cancer, members of the NUP family have also been implicated in a range of other diseases, including viral infections (11) and neurodegenerative disorders (12). For instance, alterations in certain nucleoporins have been associated with impaired immune responses and disrupted neuronal transport processes (13). However, there is limited research on the relationship between NUP153 and cancer.

With the development of public databases, pan-cancer research has become an important approach for analysing molecular features across different cancers (14). In this study, we analysed the relationship between NUP153 and multiple cancers, including NUP153 expression, mutations, diagnostic value, prognostic significance, its relationship with cellular pathways, immune environment, and drug sensitivity. Through spatial transcriptomics, we explored the expression of NUP153 in different cell types. We also focused on NUP153 expression in gastric cancer and used immunohistochemistry to analyse the expression levels of NUP153 in gastric cancer tissues and adjacent non-cancerous tissues.

## Method

### Data collection and processing

Cancer expression profile data of NUP153 from TCGA (https://portal.gdc.cancer.gov/) and GTEx (https://gtexportal.org/home/) databases were integrated using the UCEC Xena platform. The grouping of tumors and normal tissues was strictly based on the TCGA sample annotation. Data on normal tissue and cancer cell line protein expression were obtained from the Human Protein Atlas (HPA, https://www.proteinatlas.org/). The frequency of somatic mutations was assessed using the cBioPortal (https://www.cbioportal.org/) platform, and pan-cancer analysis was performed using the SangerBox 3.0 tool (https://sangerbox.com/). The relationship between NUP153 expression and the tumor immune microenvironment was explored using seven immune infiltration algorithms from the TIMER 2.0 database (https://timer.cistrome.org/). Chemotherapy data were sourced from the GDSC (https://www.cancerrxgene.org/), CTRP (https://portals.broadinstitute.org/ctrp/), and PRISM databases (15).

### Expression and variation analysis

Wilcoxon signed-rank tests were used to compare the differences in NUP153 mRNA expression between tumour and normal tissues. Paired sample analyses were also performed using the Wilcoxon signed-rank test. Anatomical visualisation of NUP153 tissue expression profiles was performed using the gganatogram R package, with further validation of transcriptomic and proteomic expression using the GEO and CPTAC databases. The pROC package was used to evaluate the diagnostic performance of NUP153 as a pan-cancer biomarker, while the mutation profile of NUP153 was retrieved from the cBioPortal platform (16).

### Survival and clinical outcome analysis

The relationship between NUP153 expression and clinical prognosis indicators, including overall survival (OS), disease-specific survival (DSS), progression-free interval (PFI), and disease-free interval (DFI), was analysed using the survival and survminer R packages. Kaplan-Meier survival curves and univariate Cox proportional hazards regression models were combined to identify the role of NUP153 as either a protective or risk factor for prognosis, and high-confidence survival analysis maps were generated.

### Pathway and mechanism of action analysis

Tumour samples were divided into high-expression (top 30%) and low-expression (bottom 30%) groups based on NUP153 expression levels. Gene set enrichment analysis (GSEA) was conducted to explore differential regulatory patterns of 50 characteristic gene sets and 83 metabolic gene sets across 33 malignancies. The GSVA R package was used to standardise and analyse 14 functional genomic signatures using z-scores, and Pearson correlation coefficients were calculated to assess the statistical correlation between each gene set’s z-score and NUP153 expression, identifying gene regulatory networks related to NUP153 expression.

### Tumour immune microenvironment

Spearman rank correlation analysis was employed to assess the stem cell characteristics of pan-cancer samples. Immune cell infiltration scoring and tumour purity were evaluated using seven algorithms (including CIBERSORT, CIBERSORT-ABS, EPIC, MCP-COUNTER, quanTIseq, TIMER, and xCELL) (17). Systematic validation of the regulatory relationship between NUP153 expression and immune-related gene sets (including MHC molecules, chemokine receptors, immune stimulators/inhibitors) was performed to elucidate its role in tumour immune microenvironment remodelling (16).

### NUP153 analysis in the gastric cancer single-cell dataset

Using the TISCH2 database (http://tisch.comp-genomics.org/) (18), the GSE134520 dataset was subjected to multi-omics correlation analysis via the Sparkle platform (https://grswsci.top/) (19). The Kruskal-Wallis H test was used to assess the expression heterogeneity of NUP153 in gastric cancer single-cell subpopulations. Cells were divided into positive and negative expression groups based on NUP153 expression status, and differential analysis between these groups was performed using the limma package. The AUCell algorithm was employed to score the activity of pathways related to immune regulation, metabolic reprogramming, signal transduction, cell proliferation, apoptosis, and mitochondrial function, analysing NUP153-dependent cellular functional regulatory networks (20).

### Spatial transcriptomic analysis of NUP153 and immune cells

Spatial transcriptomic analysis was conducted in conjunction with the Sparkle platform (https://grswsci.top/) (19) and the SpatialTME tool (https://www.spatialtme.yelab.site/). The Spotlight algorithm was used to analyse the major cell type composition of each spatial micro-region, and visualisation was carried out using the SpatialDimPlot function in the Seurat package. Gene expression landscapes were visualised using the SpatialFeaturePlot function, and spatial correlation analysis was performed using the Mantel test. Finally, spatial network visualisation was achieved using the LINCET package, systematically analysing the association mechanisms between NUP153 expression and immune cell spatial distribution (15).

### Quantitative real-time PCR

Total RNA was extracted from Gastric cancer tissues from real patients (n=3) using TRIzol reagent (15596026CN, Thermo Fisher, USA) according to the manufacturer’s instructions, and amplified using SYBR Green Master Mix(RR820A, Takara, China). Relative mRNA expression was calculated via the 2^(-ΔΔCt) method with β-actin as the reference. The upstream and downstream primers for NUP153 and β-actin were designed by Sangon(Shanghai). The primers used were as follows:

NUP153: Forward: 5’-AGCTGAGGAGATGGAGGATG-3’, Reverse: 5’-TTCAGCTCCTCCAGCTCCTC-3’;
β-actin: Forward: 5’-CTCTTCCAGCCTTCCTTCCT-3’, Reverse: 5’-AGCACTGTGTTGGCGTACAG-3’.

### Immunohistochemical analysis

This study used immunohistochemistry to examine the expression of NUP153 in gastric cancer tissues and corresponding adjacent non-cancerous tissues. The antibody used was NUP153 (14189-1-AP, Proteintech, China). Tissue samples were obtained from the People’s Hospital of Guizhou Province, including gastric cancer and adjacent non-cancer tissues (n=3). Immunohistochemical staining was performed following standard protocols, and ImageJ (v1.8) was used for data analysis. Graphs and statistical analyses were generated using Prism (v10.4.2) to evaluate the expression intensity and distribution of NUP153.

## Results

### NUP153 expression in cancer

**Figure 1:**
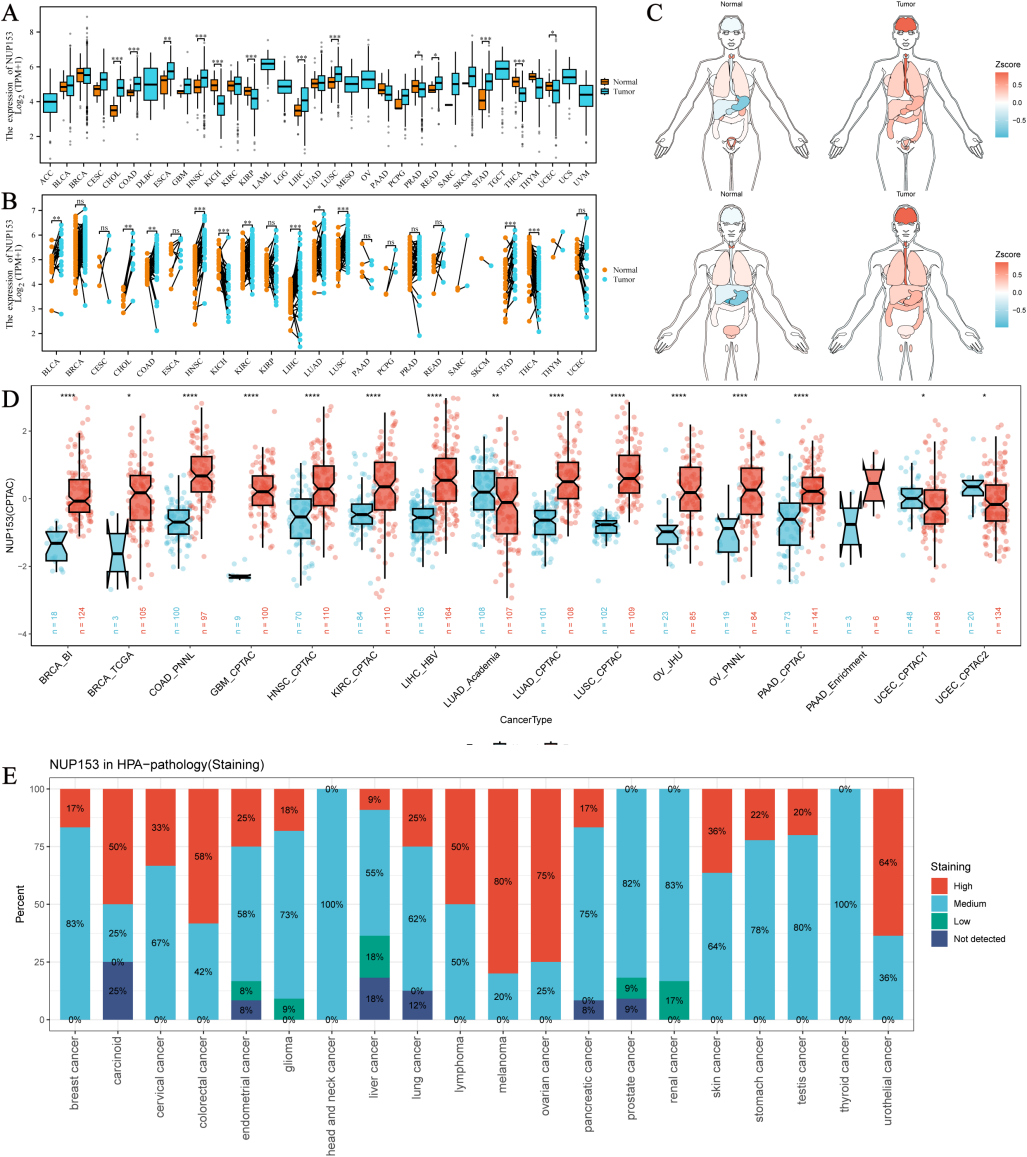
Expression of NUP153 in Tumour and Normal Tissues Across Various Cancers. **(A)** Expression of NUP153 in tumour and normal tissues from TCGA database. **(B)** Expression of NUP153 in paired tumour and adjacent normal tissues. **(C)** Organ-specific expression of NUP153 in tumour and normal tissues. **(D)** Cross-validation of NUP153 expression in tumour and normal tissues from the CPTAC database. **(E)** Immunohistochemical results of NUP153 expression from the HPA database. *p < 0.05; **p < 0.01; ***p < 0.001; ****p < 0.0001.

Through multi-dimensional analysis of unpaired tumour samples and paired tumour-normal samples from the TCGA database, we systematically analysed the expression profile of NUP153 in 33 types of cancer and physiological states. In cholangiocarcinoma (CHOL), colorectal adenocarcinoma (COAD), head and neck squamous cell carcinoma (HNSC), lung squamous cell carcinoma (LUSC), and stomach adenocarcinoma (STAD, a type of gastric cancer), NUP153 expression was higher than in normal human tissues, while in kidney chromophobe (KICH) and thyroid cancer (THCA), expression was significantly downregulated (**Figures 1A, B**). The organ-specific expression map showed that NUP153 levels were relatively low in the liver and stomach under normal conditions, but markedly elevated in the brain, oesophagus, and stomach in cancer tissues (**Figure 1C**). Cross-validation with the CPTAC database confirmed that NUP153 expression was higher in COAD, HNSC, and LUSC than in normal tissues (**Figure 1D**). Immunohistochemistry analysis showed that the protein exhibited moderate to high nuclear/nuclear membrane immunoreactivity in various cancers, with staining intensity positively correlated with protein abundance (**Figure 1E**).

### NUP153 mutations in cancer

**Figure 2:**
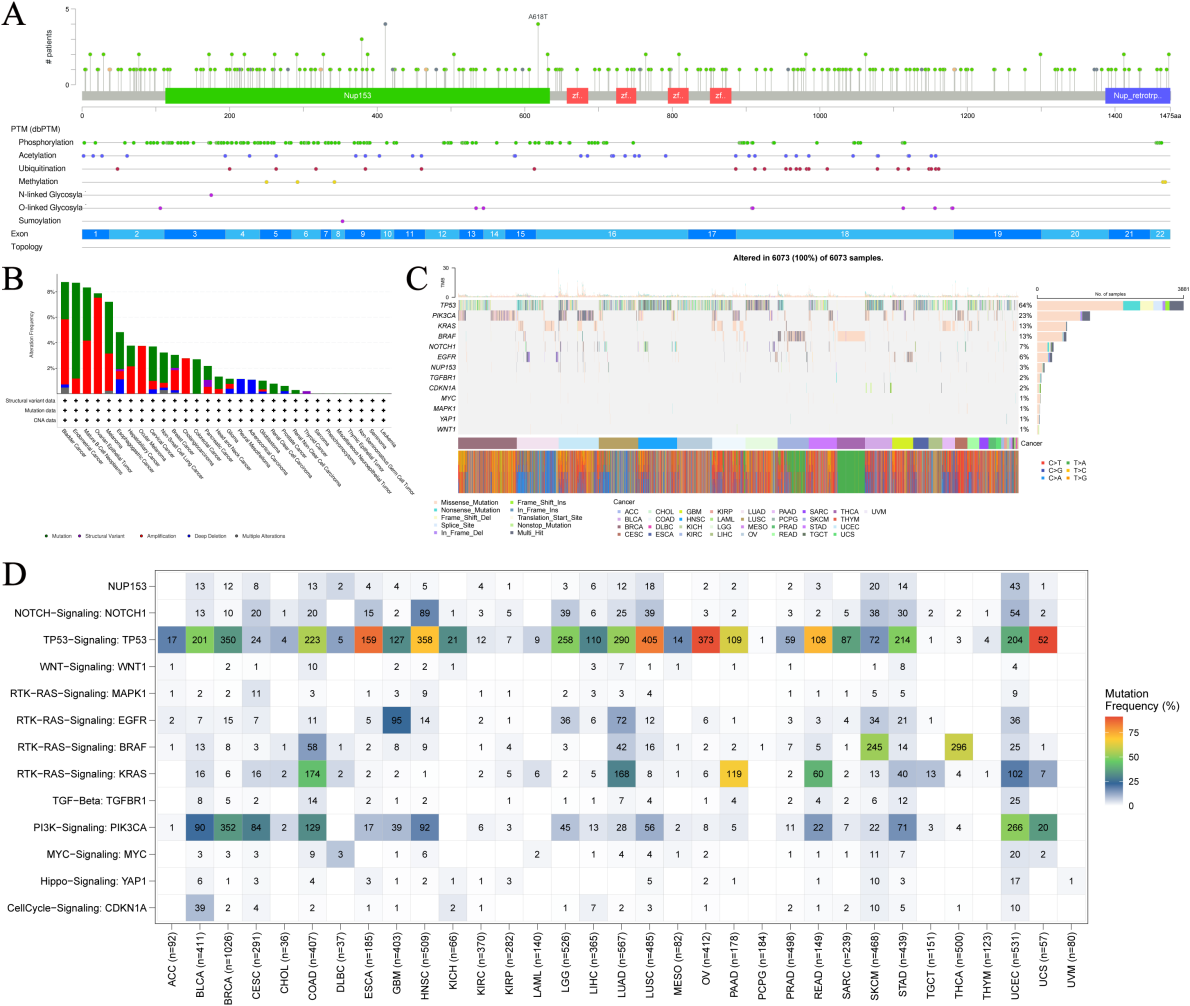
Mutation hotspots and frequency of NUP153 in various cancers. **(A)** Spatial distribution of NUP153 mutation hotspots. **(B)** Mutation frequency and types of NUP153 in cancer. **(C)** Distribution and count of NUP153 genetic alterations in cancer. **(D)** Distribution of NUP153 and other cancer-related signals in cancer.

To further understand the relationship between NUP153 expression and cancer, we analysed NUP153 mutations in cancer. **Figure 2A** shows the hotspot mutation distribution of NUP153. The mutation types of NUP153 differ across cancers, with bladder urothelial carcinoma (BLCA), uterine endometrioid carcinoma (UCEC), and mature B-cell lymphoma showing the highest mutation frequencies (**Figure 2B**). The most common mutation types were amplification and point mutations. Analysis of somatic mutations in 6,073 TCGA samples, focusing on common cancer-related genes, is presented as a heatmap in **Figure 2C**. Nonsense mutations were the predominant type of NUP153 mutations. **Figure 2D** visually displays the mutation frequency of cancer-related genes in cancer, with NUP153 showing the highest tumour mutation burden (TMB) in UCEC.

### NUP153 diagnostic value in cancer

**Figure 3:**
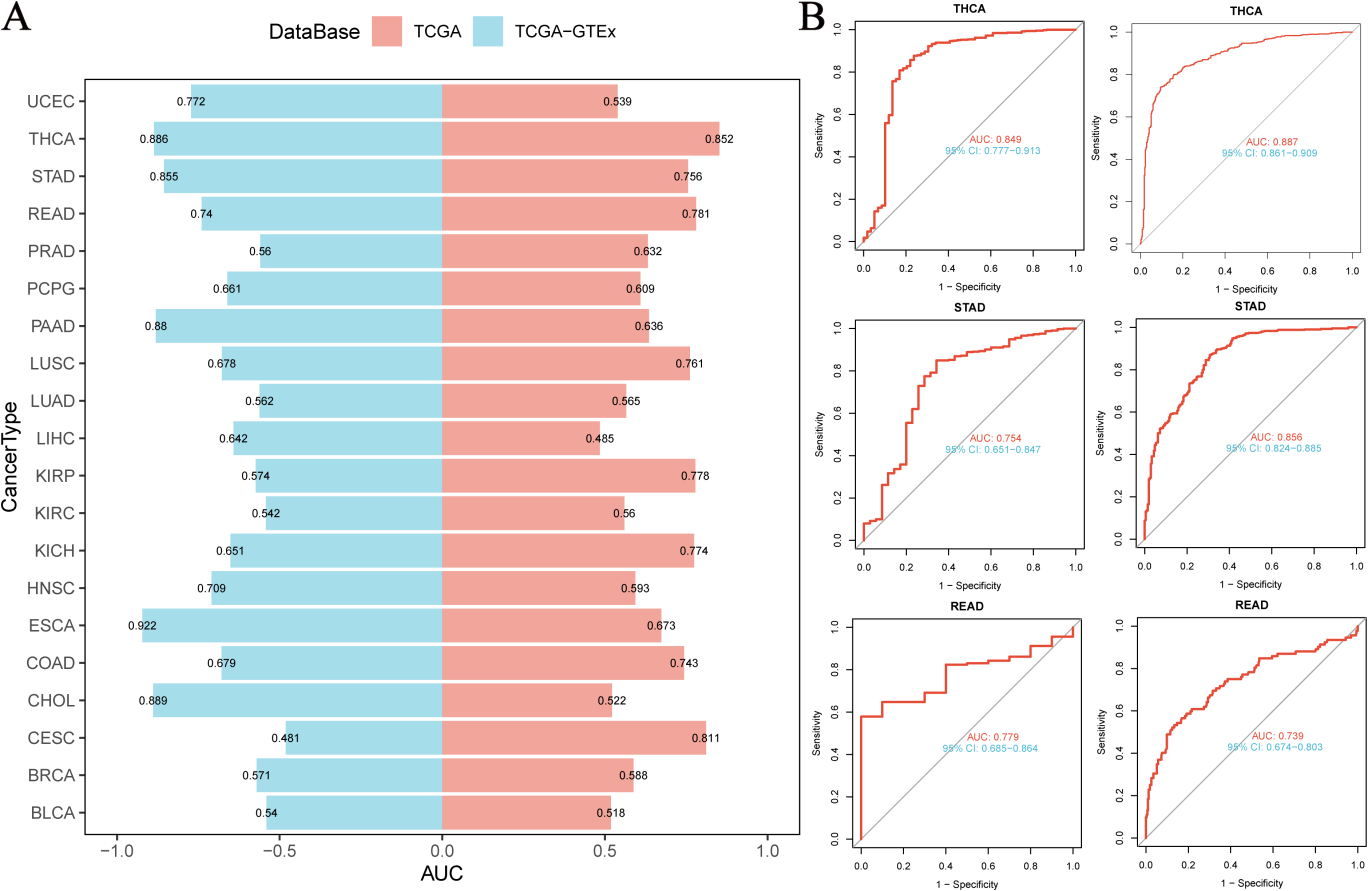
Diagnostic value of NUP153 in various cancers. **(A)** AUC values of NUP153 in the TCGA and TCGA-GTEx databases. **(B)** ROC curve of NUP153 in thyroid cancer (THCA), gastric cancer (STAD), and rectal cancer (READ).

Based on the integrated analysis of the TCGA pan-cancer cohort and the TCGA-GTEx normal tissue database, we systematically evaluated the diagnostic efficacy of NUP153 as a cancer biomarker. Through ROC curve construction, we found that in THCA, STAD, and rectal cancer (READ), the area under the curve (AUC) was above the threshold of 0.7 (**Figure 3A**), indicating a moderate diagnostic potential. Further analysis of diagnostic performance curves showed a significant correlation between NUP153 expression levels and the sensitivity and specificity in distinguishing cancer from non-cancer samples (**Figure 3B**), supporting its potential as an auxiliary diagnostic biomarker.

### NUP153 expression and prognosis in cancer

**Figure 4:**
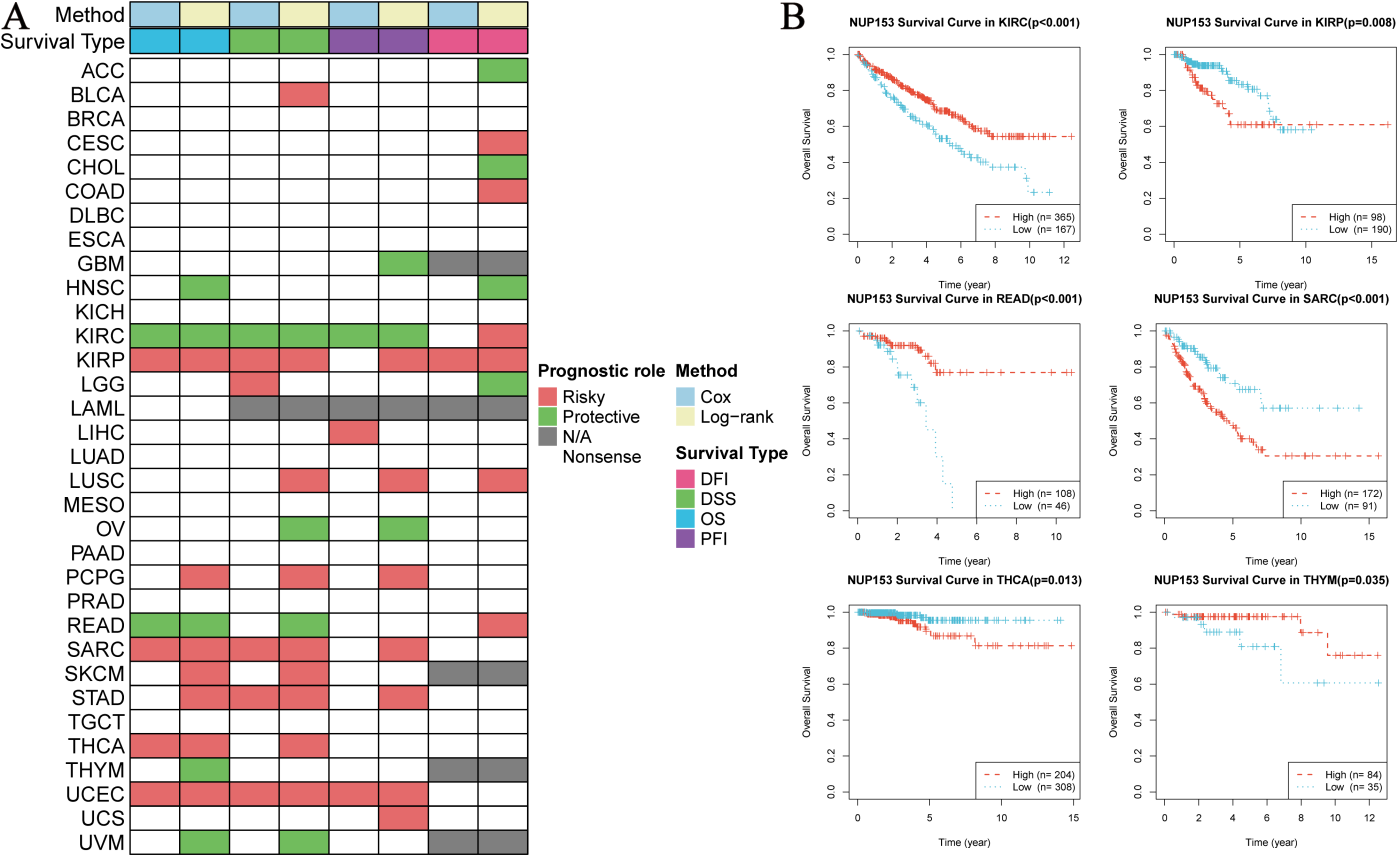
Correlation between NUP153 expression and prognosis in various cancers. **(A)** Correlation of NUP153 expression with overall survival (OS), disease-specific survival (DSS), progression-free interval (PFI), and disease-free interval (DFI) in different cancers. **(B)** Kaplan-Meier survival curves showing the correlation between NUP153 expression and OS in KIRC, KIRP, READ, SARC, THCA, and THYM.

To explore the clinical prognostic value of NUP153 expression, we assessed the relationship between NUP153 expression and the prognosis indicators OS, DSS, PFI, and DFI using log-rank tests and univariate survival analysis. The results showed that NUP153 expression was significantly correlated with the prognosis of multiple cancers and could serve as an independent risk or protective factor. Specifically, in kidney in renal clear cell carcinoma(KIRC), NUP153 was a protective factor for prognosis, whereas in renal papillary carcinoma (KIRP), sarcoma (SARC), and UCEC, NUP153 was a risk factor for prognosis (**Figure 4A**). We then plotted the Kaplan-Meier (KM) survival curves for OS in KIRC, READ, and THCA, showing that high NUP153 expression correlated with better OS in KIRC, READ, and THYM, while it was associated with worse OS in KIRP, SARC, and THCA (**Figure 4B**).

### NUP153 expression and tumour-related pathways

**Figure 5:**
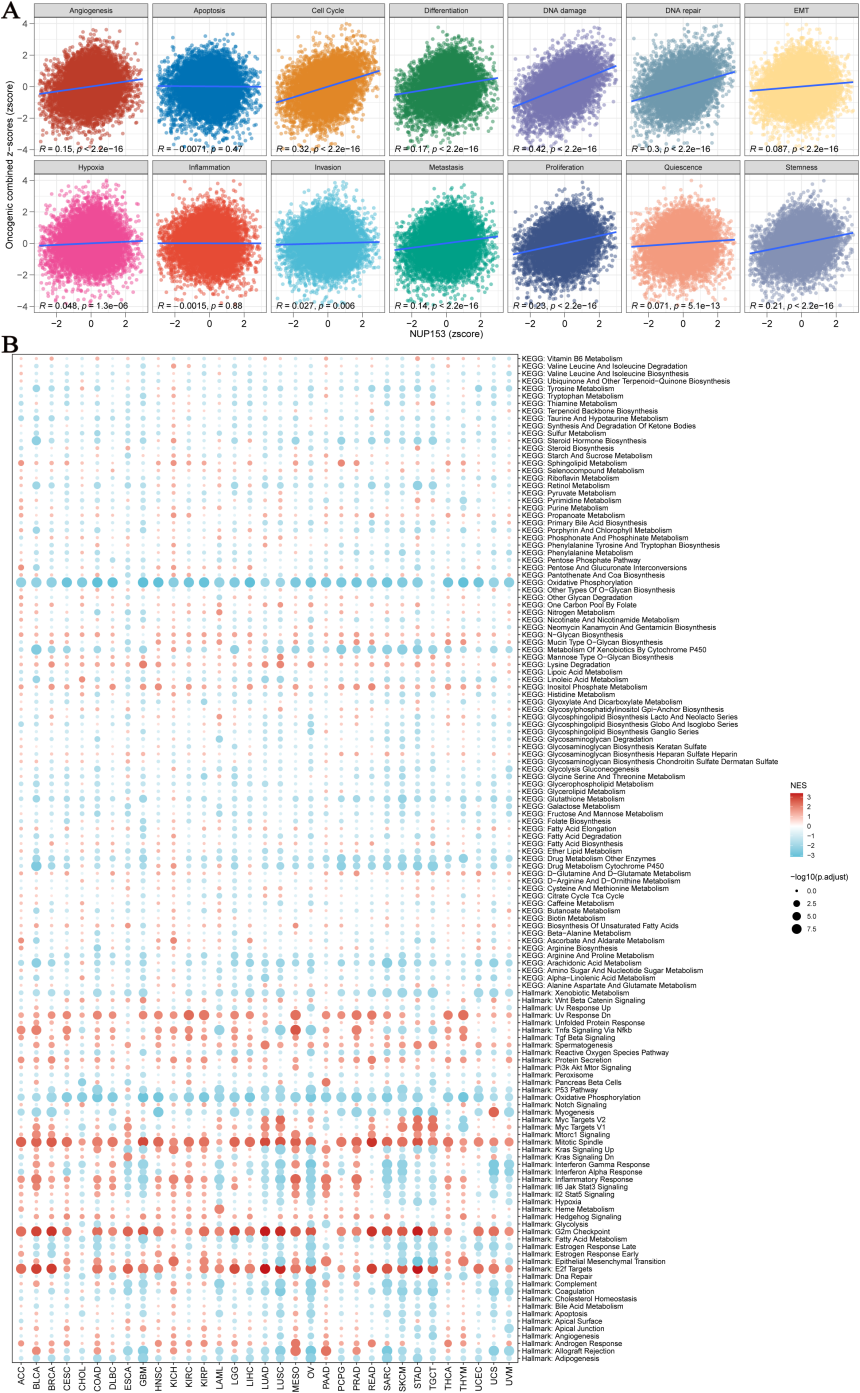
Pathway analysis and potential mechanism analysis related to NUP153 expression. **(A)** Correlation between NUP153 mRNA expression and 14 malignant features of tumours. **(B)** Enrichment differences of NUP153 in 50 HALLMARK and 83 metabolic gene sets.

We used an integrated feature gene expression analysis method to calculate the gene sets related to 14 cancer-associated functional states, and assessed the activity levels of each pathway using the combined z-score normalization values. These 14 functional gene sets include: angiogenesis, apoptosis, cell cycle, differentiation, DNA damage, DNA repair, epithelial-mesenchymal transition (EMT), hypoxia, inflammation, invasion, metastasis, proliferation, quiescence, and stemness. Pearson correlation analysis showed that NUP153 expression was significantly and positively correlated with the activity of cell cycle regulation and DNA damage response pathways (**Figure 5A**). Further GSEA of the top 30% and bottom 30% of NUP153 expression samples revealed significant enrichment of oxidative phosphorylation and cytochrome P450-mediated xenobiotic metabolism pathways in the low-expression group, while mitotic spindle assembly, G2/M checkpoint, and E2F target gene pathways were significantly enriched in the high-expression group (**Figure 5B**). These findings suggest that NUP153 may be involved in regulating multiple cancer-related pathways and metabolic processes.

### NUP153 and functional proteins and cell pathways

**Figure 6:**
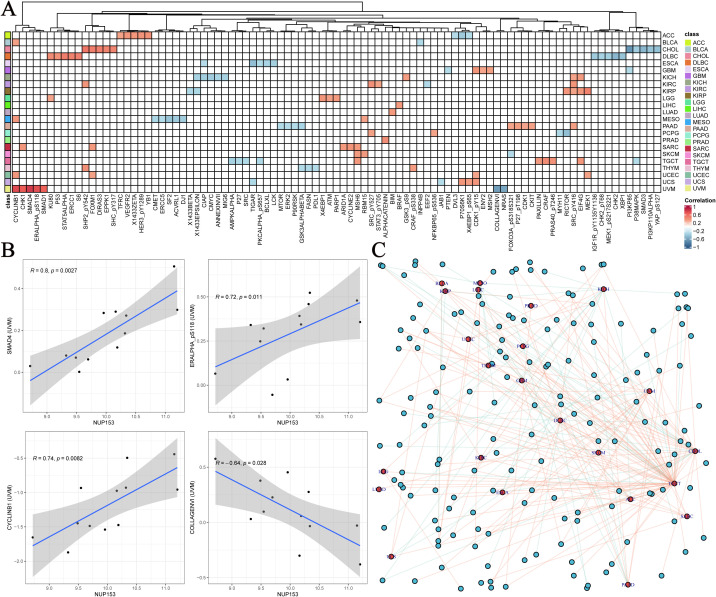
Functional protein network related to NUP153 expression in various cancers. **(A)** Top five functional proteins most correlated with NUP153 expression in various cancer types. **(B)** Significant functional proteins of NUP153 in uveal melanoma(UVM). **(C)** Network of relationships between NUP153 and functional proteins across different cancer types.

To further explore the functional network of NUP153, we used the TCPA database to analyse its association with tumour-related functional proteins. **Figure 6A** shows the distribution of the top five correlated functional proteins in various tumour tissues. In uveal melanoma (UVM), NUP153 expression was significantly positively correlated with SMAD4, CYCLINB1, and phosphorylated ERα (ERALPHA-ps118) protein levels, and negatively correlated with type VI collagen (collagen vi) and NRAS protein levels. **Figure 6B** displays the most significant functional proteins of NUP153 in UVM. We then constructed a relationship network between cancer and functional proteins (**Figure 6C**).

### NUP153 and immune-related genes and immune infiltration levels

**Figure 7:**
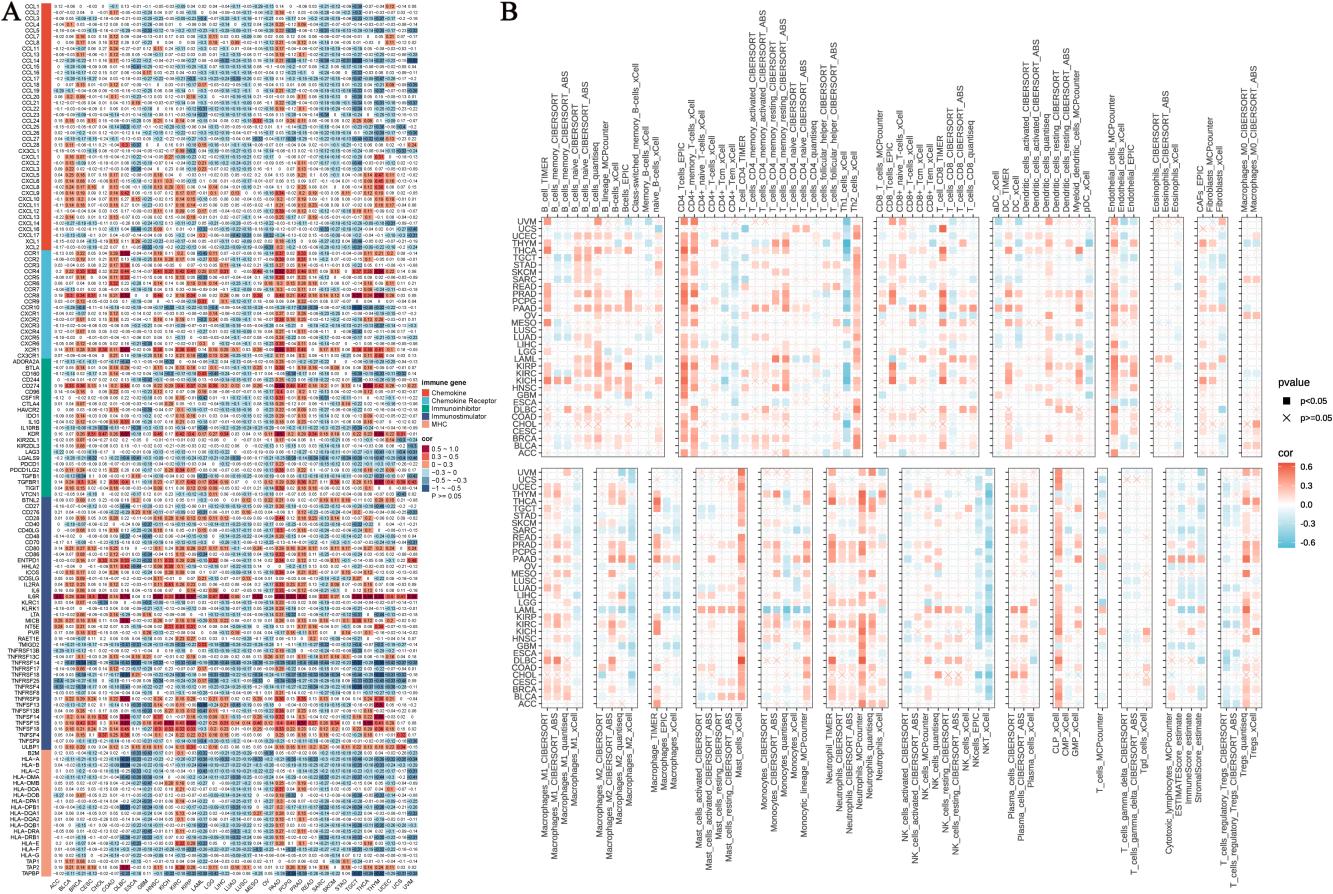
NUP153 expression and its relationship with immune-related genes and immune infiltration. **(A)** Correlation between NUP153 expression and chemokine family genes, chemokine receptor family genes, immune checkpoint inhibitors, immune stimulators, and MHC complex genes. **(B)** Relationship between NUP153 expression and immune infiltration levels in the tumour microenvironment.

We systematically evaluated the regulatory role of NUP153 in the tumour immune microenvironment. Heatmap analysis revealed the complex interaction patterns between NUP153 expression and chemokine family genes, chemokine receptor family genes, immune checkpoint inhibitors, immune stimulators, and MHC complex gene expressions. Notably, in lung adenocarcinoma (LUAD) and LUSC, NUP153 expression was significantly negatively correlated with immune-related genes; whereas, in pancreatic ductal adenocarcinoma (PAAD) and prostate cancer (PRAD), a positive correlation was observed (**Figure 7A**). Immune infiltration analysis, cross-validated using seven independent algorithms, demonstrated a significant association between NUP153 expression and immune cell infiltration levels within the tumour microenvironment. Pan-cancer analysis revealed that in most cancers, NUP153 expression was positively correlated with tumour-associated macrophage (TAM) infiltration levels (**Figure 7B**). The results of these algorithms present a high degree of consistency.

### NUP153 and chemotherapy in cancer

**Figure 8:**
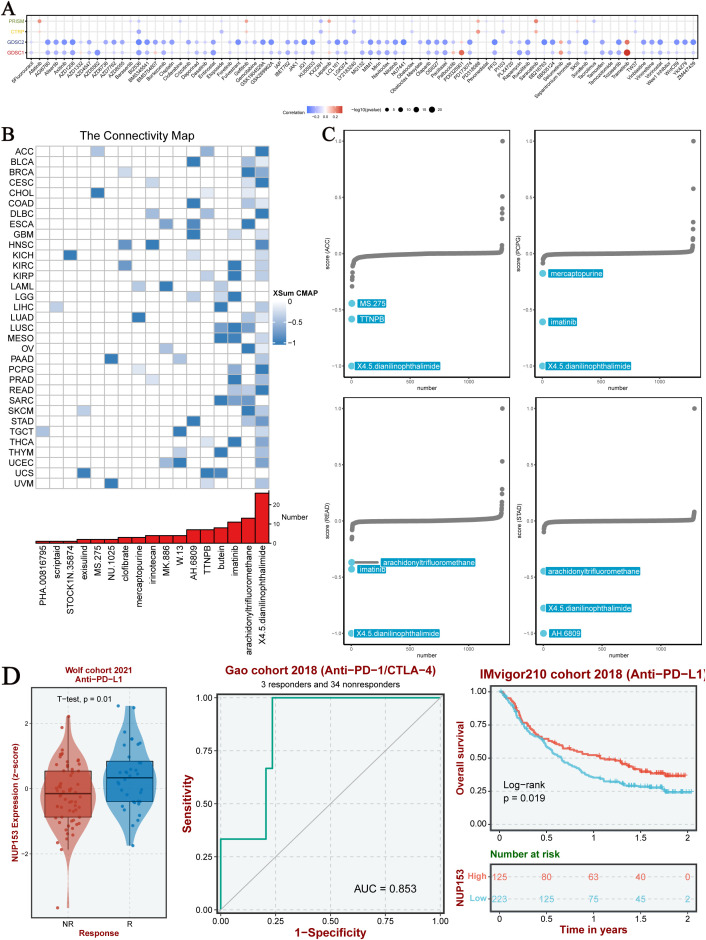
Chemotherapy sensitivity and NUP153 expression in pan-cancer analysis. **(A)** Correlation between NUP153 mRNA expression and chemotherapy sensitivity from the PRISM, GDSC1, GDSC2, and CTRP databases. **(B)** Identification of NUP153-targeted compounds through Cmap analysis. **(C)** Clinical relevance of drugs targeting NUP153 and their effects on cancer cells.

To explore the clinical translational value of NUP153, we integrated data from the PRISM, CTRP, GDSC1, and GDSC2 drug sensitivity databases for analysis. The results indicated that in both the GDSC1 and GDSC2 databases, NUP153 expression was significantly negatively correlated with the sensitivity to most chemotherapy drugs (**Figure 8A**). Compound-gene perturbation analysis via the Cmap database (**Figure 8B**) revealed that 4,5-diamino-2-phthalimidobenzene (X4.5dianilinophthalimide) and Imatinib significantly inhibited NUP153 activity in multiple cancers. Preclinical validation showed that X4.5dianilinophthalimide significantly suppressed NUP153 activity in adrenal cortical carcinoma (ACC), pheochromocytoma and paraganglioma (PCPG), and READ, while Imatinib exhibited similar inhibitory effects in STAD (**Figure 8C**). Further clinical cohort analysis demonstrated that patients with high NUP153 expression had significantly higher response rates to immunotherapy, and we assessed the potential of NUP153 as a predictor of immunotherapy response. Survival analysis revealed that high NUP153 expression was associated with significantly prolonged survival (**Figure 8D**).

### Spatial transcriptomics

**Figure 9:**
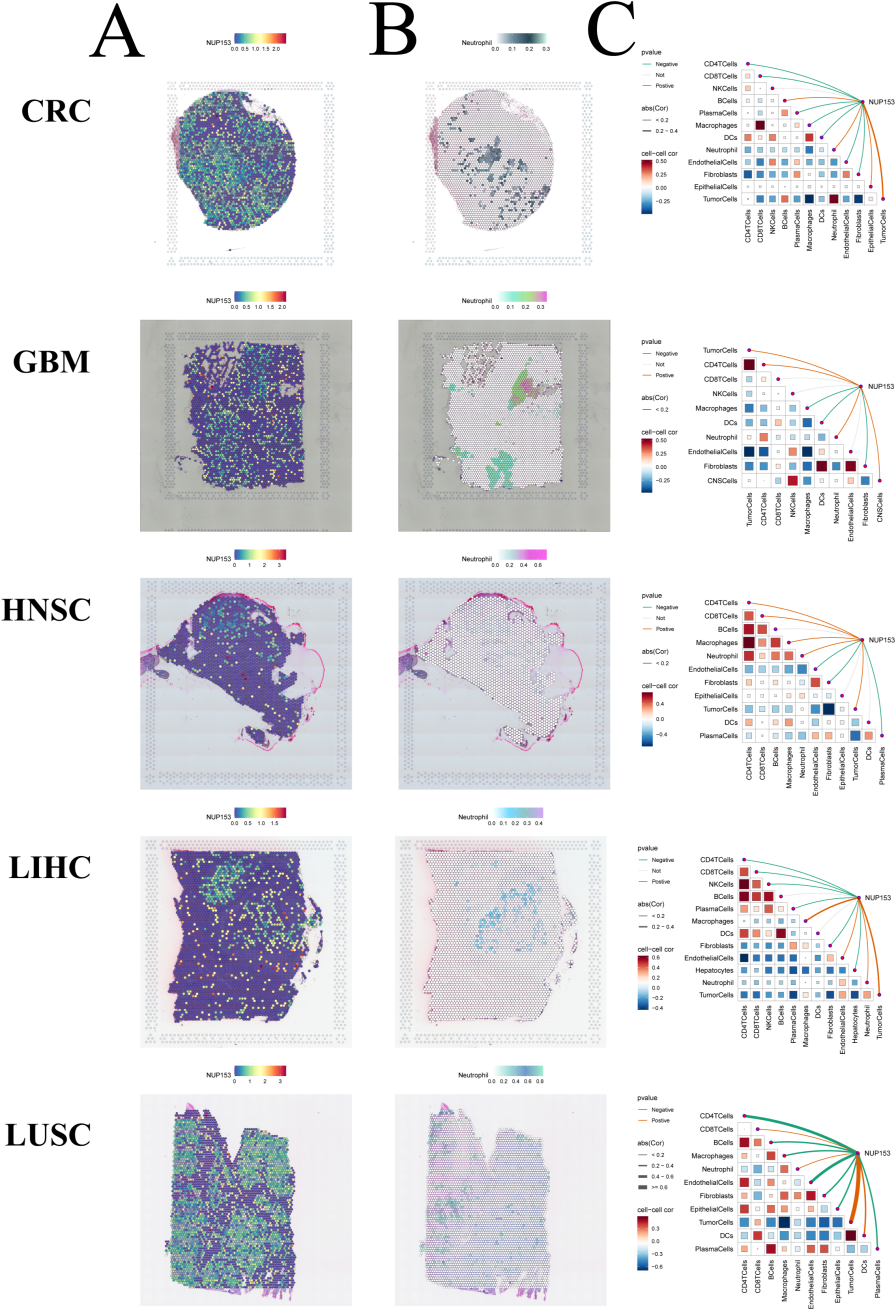
Spatial transcriptomics analysis of NUP153 expression and neutrophil infiltration in the tumour microenvironment. **(A)** NUP153 expression levels in spatial transcriptomic data. **(B)** Spatial distribution of neutrophils in the tumour microenvironment. **(C)** Correlation between cell content and NUP153 expression levels across all micro-regions.

Spatial transcriptomics allows for simultaneous acquisition of both cellular spatial location information and gene expression data, providing an important research tool for understanding tissue cell functions and microenvironment interactions. Thus, we visualised the gene expression landscapes of each micro-region using spatial transcriptomic data. We utilised the SpatialFeaturePlot function from the Seurat package to visualise the enrichment scores for each cell type. The deeper the colour, the higher the cell type content in the spot. We also analysed the correlation between NUP153 expression levels and cell content across all the spots (**Figures 9A-C**). These results were highly consistent with our findings from transcriptomic data: in colorectal cancer(CRC), LIHC, HNSC, GBM, and LUSC, NUP153 expression positively correlated with neutrophil distribution. These results suggest that NUP153 may play a role in regulating tumour immune cell infiltration.

### NUP153 in gastric cancer single-cell data

**Figure 10:**
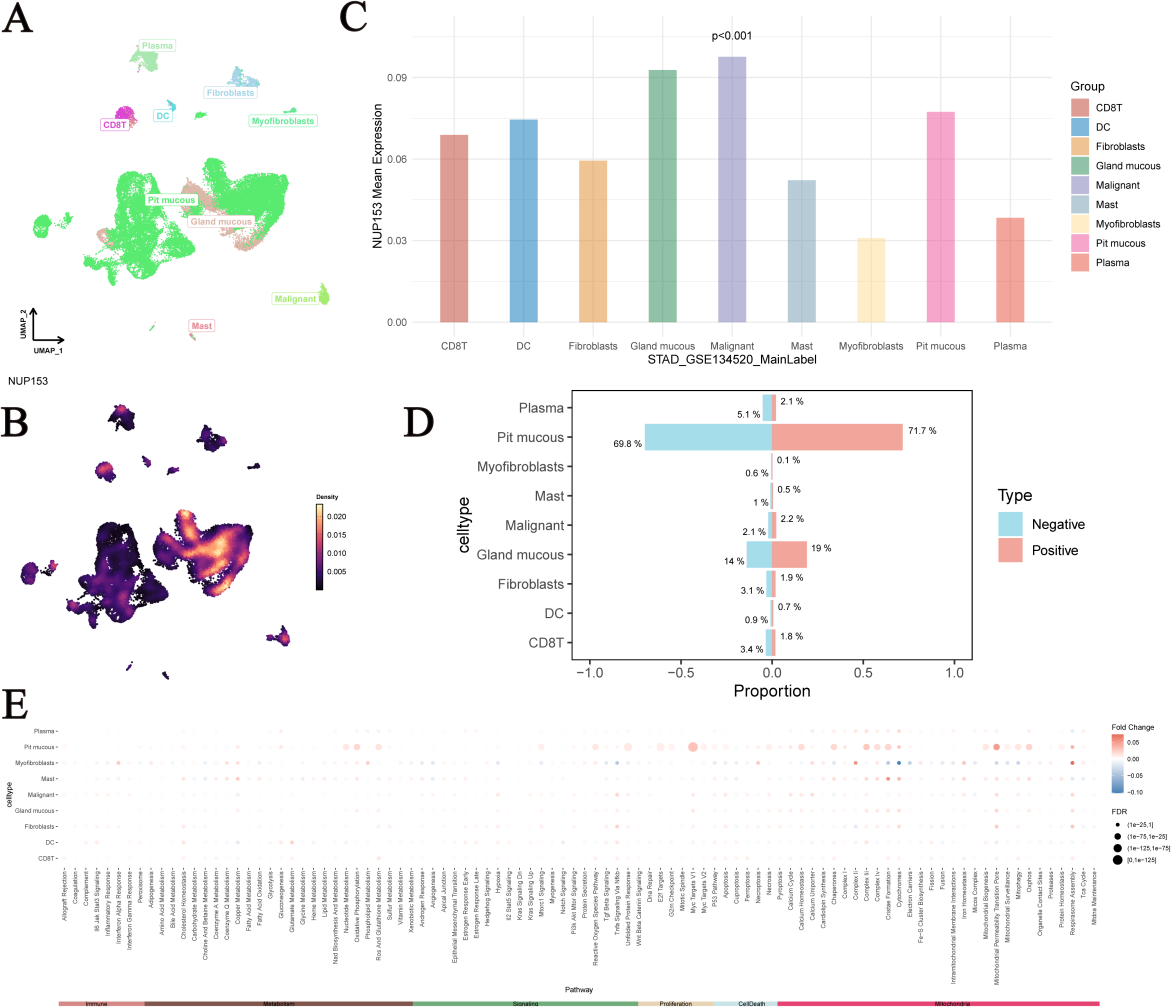
Single-cell analysis of NUP153 expression in gastric cancer subpopulations. **(A)** Distribution of single-cell data from the GSE134520 dataset after dimensionality reduction. **(B)** NUP153 expression levels in single cells. **(C)** Differences in NUP153 expression across different cell types. **(D)** Proportions of different cell types in the NUP153-positive and NUP153-negative groups. **(E)** Differences in pathway scores between NUP153-negative and NUP153-positive groups.

UMAP analysis showed the distribution of single-cell data from the GSE134520 dataset after dimensionality reduction, with different cell types separated based on unique expression patterns (**Figure 10A**). We visualised NUP153 expression levels in single cells (**Figure 10B**) and further assessed the differences in NUP153 expression across different cell types (**Figure 10C**). Based on whether cells expressed NUP153, we divided all cells into positive and negative expression groups and calculated the proportions of different cell types in both groups (**Figure 10D**). NUP153 showed too many differences between the positive and negative groups. Additionally, we assessed the activity scores of immune, metabolic, signalling, proliferation, apoptosis, and mitochondrial-related pathways and compared the score differences between the NUP153 negative and positive expression groups (**Figure 10E**). The results showed that in mucus cells, the NUP153-positive group had significantly higher activity scores on proliferation, metabolism and mitochondria-related biological pathways. Especially Myc Targats V1 and Complex I. These findings reveal the potential mechanistic role of NUP153 in gastric cancer.

### Validation of NUP153 expression in gastric cancer

**Figure 11:**
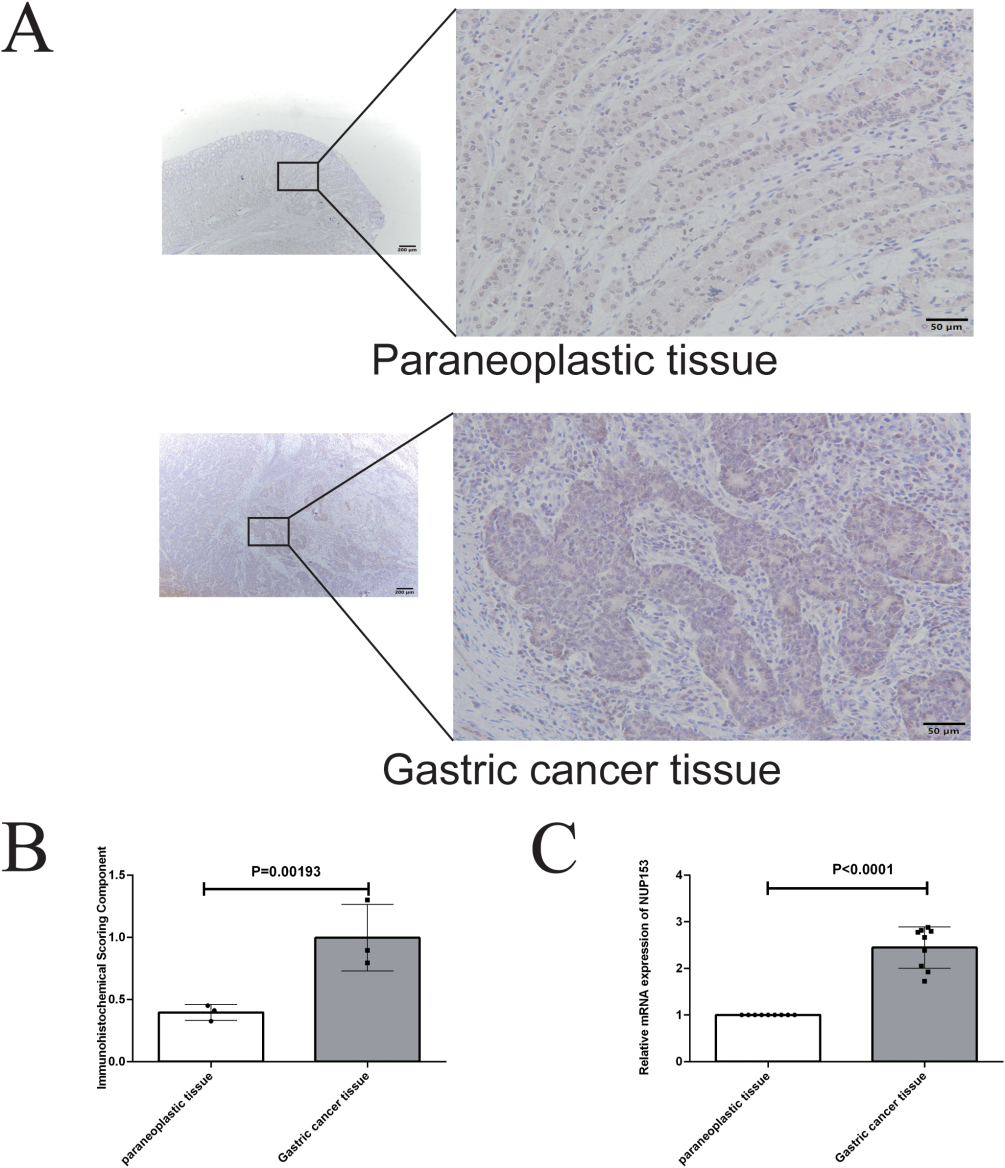
Validation of NUP153 expression in gastric cancer tissues and adjacent non-cancerous tissues. **(A, B)** Protein expression levels of NUP153 in gastric cancer tissues and adjacent non-cancerous tissues. **(C)** mRNA expression levels of NUP153 in gastric cancer tissues and adjacent non-cancerous tissues.

To further validate the association between NUP153 and gastric cancer, we conducted an external validation using human gastric cancer tissue samples. Immunohistochemistry was employed to detect NUP153 protein expression and compare its levels between gastric cancer tissues and adjacent non-cancerous tissues. The results (**Figures 11A, B**) showed that NUP153 expression was significantly higher in gastric cancer tissues than in adjacent non-cancerous tissues (*p* < 0.05). Additionally, RT-qPCR was used to detect NUP153 mRNA expression, confirming that its levels were significantly higher in gastric cancer tissues compared to non-cancerous tissues (*p* < 0.05) (**Figure 11C**).

## Discussion

Previous studies have confirmed that NUP153 is involved in the regulation of nucleocytoplasmic transport and mitosis (21, 22). This study focuses on investigating the relationship between NUP153 expression levels and cancer. Specifically, the study primarily examines the expression characteristics of NUP153 in gastric cancer cells and further verifies the protein expression of NUP153 in gastric cancer tissues through experiments.

To gain a broader understanding of its role in cancer, we first analysed the expression pattern of NUP153 across multiple cancer types and normal tissues. The results revealed significant expression heterogeneity among cancers.It is highly expressed in CHOL, COAD, and HNSC, suggesting its potential as a candidate biomarker. Furthermore, the expression profile of NUP153 varies across organs, with lower expression in the liver and stomach in normal tissues, while higher expression in gastric and oesophageal cancer tissues may reflect functional activation during carcinogenesis, or be associated with malignant tumour characteristics such as metastatic potential. The high expression of NUP153 in the brain, oesophagus, and gastric cancer tissues likely indicates a stronger dependence of these organs’ tumours on NUP153 functions (23), further supporting its organ-specific characteristics (24). Our study further suggests that NUP153 tends to exhibit strong immunoreactivity in the nucleus and nuclear membrane across a range of cancers. This observation may reflect a potential involvement of nuclear pore complex proteins in modulating nucleocytoplasmic transport—for instance, facilitating the nuclear import of oncogenic proteins or the export of tumour suppressors—which could play a role in tumorigenesis. These findings are in line with some previously reported studies (7, 25). These differences in expression levels may stem from diverse regulatory mechanisms such as epigenetic modifications, transcriptional control, or interactions with tumour-specific signalling pathways (26). Understanding these mechanisms could offer new perspectives on how NUP153 contributes to tumour heterogeneity.

Early cancer diagnosis is one of the key methods for improving cancer patient prognosis, and pan-cancer analysis provides an effective strategy for discovering novel biomarkers (27). This study found that NUP153 exhibits AUC values greater than 0.7 in THCA, STAD, and READ, indicating its significant diagnostic potential and suggesting that it could serve as a candidate diagnostic biomarker. This study also revealed a significant correlation between NUP153 expression and cancer prognosis. Specifically, high NUP153 expression was associated with improved survival in KIRC, while it was associated with reduced survival in KIRP, SARC, and UCEC. This result suggests that NUP153 could serve as a prognostic biomarker for certain cancers, providing reference for clinical decision-making. This finding is partly consistent with recent research results (24, 28). Given its significant correlations with patient prognosis across multiple cancer types, NUP153 holds potential as a clinically relevant biomarker. Its integration into diagnostic or prognostic frameworks may enhance the precision of early detection and personalised treatment strategies.

Our study also found that NUP153 expression is positively correlated with the cell cycle and DNA damage response, suggesting that upregulated NUP153 expression may promote cell proliferation and induce cell damage, possibly related to membrane stability during the S phase (29). Furthermore, GSEA revealed significant enrichment of pathways related to mitotic spindle assembly, the G2/M checkpoint, and E2F target genes in the high NUP153 expression group, reinforcing the hypothesis that NUP153 may play a role in regulating cell division. These findings indicate that suppressing NUP153 expression could represent a potential strategy to inhibit tumour cell proliferation in cancers characterised by its high expression. Moreover, the widespread expression pattern of NUP153 provides valuable insights for further exploration of its involvement in tumour initiation and progression.

In addition, our study highlighted that NUP153 expression showed heterogeneous immune infiltration characteristics across different cancers. In some cancer types, NUP153 expression is positively correlated with immune gene expression, while in others, it is negatively correlated. This suggested that the expression pattern of NUP153 could potentially serve as a predictor of sensitivity to cancer immunotherapy. Specifically, cancers with active immune gene expression may be more responsive to immunotherapy, while those with lower immune gene expression may have poorer responses to immunotherapy, a theory that is increasingly being recognised by researchers in the field of immunology (30, 31).

Moreover, our analysis also showed that NUP153 expression is negatively correlated with the sensitivity to most chemotherapy drugs in the GDSC database, suggesting that high NUP153 expression may reduce cancer cell sensitivity to chemotherapy drugs. This inverse relationship suggests that NUP153 may influence drug resistance mechanisms, underscoring its potential as a predictive marker for treatment response. Further exploration could inform the development of targeted therapies or combination strategies to overcome chemoresistance. Therefore, targeting the inhibition of NUP153 expression may serve as a potential strategy based on computational evidence to enhance chemotherapy sensitivity in cancer cells. It is important to note that the link between NUP153 and chemotherapy sensitivity is based on database correlation analysis, and the precise mechanism—such as whether it influences drug efflux via nuclear translocation—remains to be confirmed through experimental studies.In our study, X4.5-diamino-2-phthalimidobenzene (X4.5dianilinophthalimide, NAPH) and Imatinib were found to inhibit NUP153 expression in most cancers, providing new experimental evidence for chemotherapy drug selection. NAPH, a potent inhibitor of amyloid β-protein fibrillation (32), has also been explored in a few studies as an experimental treatment for lung cancer (33) and bladder cancer (34). Imatinib, on the other hand, is a critically important tyrosine kinase inhibitor with anti-tumour activity, widely used in the treatment of various cancers (35, 36). These findings not only expand the known pharmacological activities of NAPH and Imatinib, but also suggest a novel drug repositioning strategy targeting NUP153 in cancer. By identifying agents capable of downregulating NUP153, our study provides a promising therapeutic direction for tumours with high NUP153 expression, and lays the groundwork for future clinical investigations into pan-cancer therapies based on NUP153 inhibition.

It is noteworthy that our results indicated that amplification and mutations are the most common types of genetic variation in NUP153. Nonsense mutations were the most common mutation type, and amplification may lead to NUP153 overexpression, while nonsense mutations may result in a loss of protein function, suggesting that NUP153 may play a dual role in cancer, either promoting or suppressing tumour development depending on the context. The high frequency of mutations in NUP153 in UCEC suggests that it may be closely related to the development and progression of this cancer. Previous studies have focused more on nucleoporin proteins such as NUP85, NUP93, NUP107, and NUP188 (37, 38), while research on NUP153 has largely centred on its role in immune regulation, particularly in the context of HIV infection. In contrast, our study provides new insight into the cancer-specific genetic alterations of NUP153 and their functional consequences. By identifying its potential oncogenic and tumour-suppressive roles, we not only expand the understanding of NUP153 beyond virology and basic cell biology, but also highlight its value as a novel focus for cancer research. This lays a foundation for future mechanistic studies and therapeutic exploration targeting NUP153 in tumours with distinct genetic profiles. These genetic alterations likely affect NUP153’s functional role in tumour progression and may account for its varied expression across cancer types. Elucidating the impact of these mutations and copy number changes may provide a mechanistic basis for its oncogenic or tumour-suppressive functions in distinct tumour contexts.

Additionally, spatial transcriptomics data analysis showed that in CRC, liver hepatocellular carcinoma (LIHC), head and neck squamous cell carcinoma (HNSC), glioblastoma (GBM), and LUSC, NUP153 expression was positively correlated with neutrophil spatial distribution. In related studies, neutrophils have been shown to promote tumour metastasis by forming neutrophil extracellular traps (NETs) (39) and contribute to poor survival outcomes in immune-related cancer patients (40, 41). Tumours with high NUP153 expression may exhibit stronger invasiveness (24). Therefore, combining our findings, targeting NUP153 could become a potential strategy to inhibit NET formation and tumour metastasis. Furthermore, our study also conducted an in-depth analysis of single-cell data from gastric cancer, comparing the biological characteristics of NUP153-positive and negative expression groups. The results showed that in mucous cells, the NUP153-positive group had significantly higher activity scores in proliferation-related biological pathways, suggesting that high NUP153 expression may be associated with enhanced proliferative activity in mucous cells. Therefore, we propose that targeting NUP153 could become a potential therapeutic strategy to specifically eliminate high-proliferation mucous cell subpopulations and delay gastric cancer progression. Notably, the high expression of NUP153 in specific cells suggests that its function is cell-state dependent, which may make it a potential marker for gastric cancer subtyping.

Finally, through experimental verification, we analysed the expression levels of NUP153 protein in gastric cancer tissues and adjacent non-cancerous tissues. The results showed that NUP153 protein expression was significantly higher in gastric cancer tissues than in the surrounding normal tissues. This finding reinforces the hypothesis that NUP153 plays a critical role in the development and progression of gastric cancer. Importantly, this experimental evidence complements the bioinformatic and pan-cancer analyses conducted earlier in our study, strengthening the link between NUP153 overexpression and malignant phenotypes. Taken together, these results suggest that NUP153 may serve not only as a biomarker for gastric cancer diagnosis or prognosis, but also as a viable molecular target for therapeutic intervention. Further exploration of NUP153-targeted strategies could contribute to the development of more effective and personalised treatments for gastric cancer.

## Limitation

This study has several limitations. First, variations in sample sources and batch effects in public databases may affect the generalisability of the findings. A larger sample size is needed to confirm the robustness of the conclusions. Second, although the expression pattern of NUP153 in gastric cancer was validated experimentally, the sample size used was limited. The underlying molecular mechanisms also remain unclear and require further investigation through functional assays. Moreover, the predictive value of NUP153 expression for immunotherapy response and chemotherapy sensitivity needs to be tested in prospective clinical studies, particularly regarding its potential as a target for combination therapy. Finally, this study did not examine the possible interactions between NUP153 and other nucleoporins. Future research could explore this through integrated multi-omics approaches to better understand its role within the nuclear pore complex.

## Conclusion

In conclusion, this study systematically explored the expression patterns and functional roles of NUP153 across various cancers through multi-omics analysis and experimental validation. We found that NUP153 is highly expressed in several cancers, including gastric cancer, and is closely linked to tumour cell proliferation, changes in the immune microenvironment, and resistance to chemotherapy. Its involvement in key biological processes such as the cell cycle and DNA damage response supports its potential as a biomarker and therapeutic target. In particular, the high expression of NUP153 in gastric cancer may be associated with tumour progression, offering a new direction for future clinical strategies. However, the precise molecular mechanisms of NUP153 in cancer remain to be further investigated to support its clinical application.

## Data Availability

Publicly available datasets were analyzed in this study. This data can be found here: The GSE134520 dataset was downloading from GEO database (https://www.ncbi.nlm.nih.gov/gds/?term=GSE134520).
